# The Current Status in Obstetrics in North Korea and Strategies for Establishing a Better Healthcare System

**DOI:** 10.3389/fpubh.2021.744326

**Published:** 2021-12-24

**Authors:** Joseph J. Noh

**Affiliations:** Department of Obstetrics and Gynecology, Samsung Medical Center, Sungkyunkwan University School of Medicine, Seoul, South Korea

**Keywords:** Democratic People's Republic of Korea, Republic of Korea, delivery of health care, obstetrics, maternal mortality, North Korea

## Abstract

The women's healthcare in North Korea is in poor condition. The present study explored the current state of women's healthcare, especially in the field of obstetrics, in the region with a number of considerations in regards to establishing a better healthcare system. Peer-reviewed journal articles and reports from intergovernmental organizations were reviewed. Data show that many healthcare facilities suffer from shortages of basic amenities. The maternal mortality ratio was 82 deaths per 100,000 live births. The leading cause of maternal death was postpartum hemorrhage. It was also found that many hospitals were unable to provide adequate obstetrical emergency care such as anticonvulsants, antibiotics, and blood products. A long-term roadmap that is sustainable with clear principles and that is not disturbed by political tensions should be established.

## Introduction

North Korea, officially known as the Democratic People's Republic of Korea (DPRK), has maintained its sovereign state since its division with South Korea in the 1960s. Since then, the nation has not been engaged in significant inter-governmental communications with other nations. The economy has been unstable and its people have suffered through many areas of society including healthcare, education, and others. The consequence of insecurities of healthcare has held enormous burden on its citizens. For example, the current status of women's healthcare in North Korea is in dire condition, incapable of providing even minimal services. State-run hospitals and clinics are supposed to provide patients with access to free medical care under the country's universal health system. However, the access to healthcare facilities with competent quality is only available to a limited number of people, and the functions of many facilities have been impaired. The purpose of the present study is to explore the current states of women's healthcare, especially in the field of obstetrics in North Korea with an emphasis on providing sustainable and effective support programs from international communities. A number of considerations in regards to aiding the nation are discussed. It is hoped that this review can stimulate the interest and to increase the awareness of the current situations of healthcare system in North Korea especially among the professionals in the field of women's healthcare in the Korean peninsula.

## Methods of Review

MEDLINE, PubMed Health, Scopus, and Google (1975 through 2018) search of the literature was performed. It was supplemented by searches for articles in Korean language as well. The search of the databases included terms “Democratic People's Republic of Korea (DPRK) and obstetrics,” “DPRK and gynecology,” “DPRK and women's healthcare,” and “DPRK and health.” Journal articles and reports from intergovernmental organizations were selected. Many news articles, anecdotes, and opinions about the healthcare system of North Korea were found, but peer-reviewed journal articles and the articles published by official intergovernmental organizations were included for objectivity. Obstetrical care and women's healthcare in general were speculated. The protocols of the study were reviewed by the institutional review board at the present institution and were given an exempt determination.

**Figure 1:**
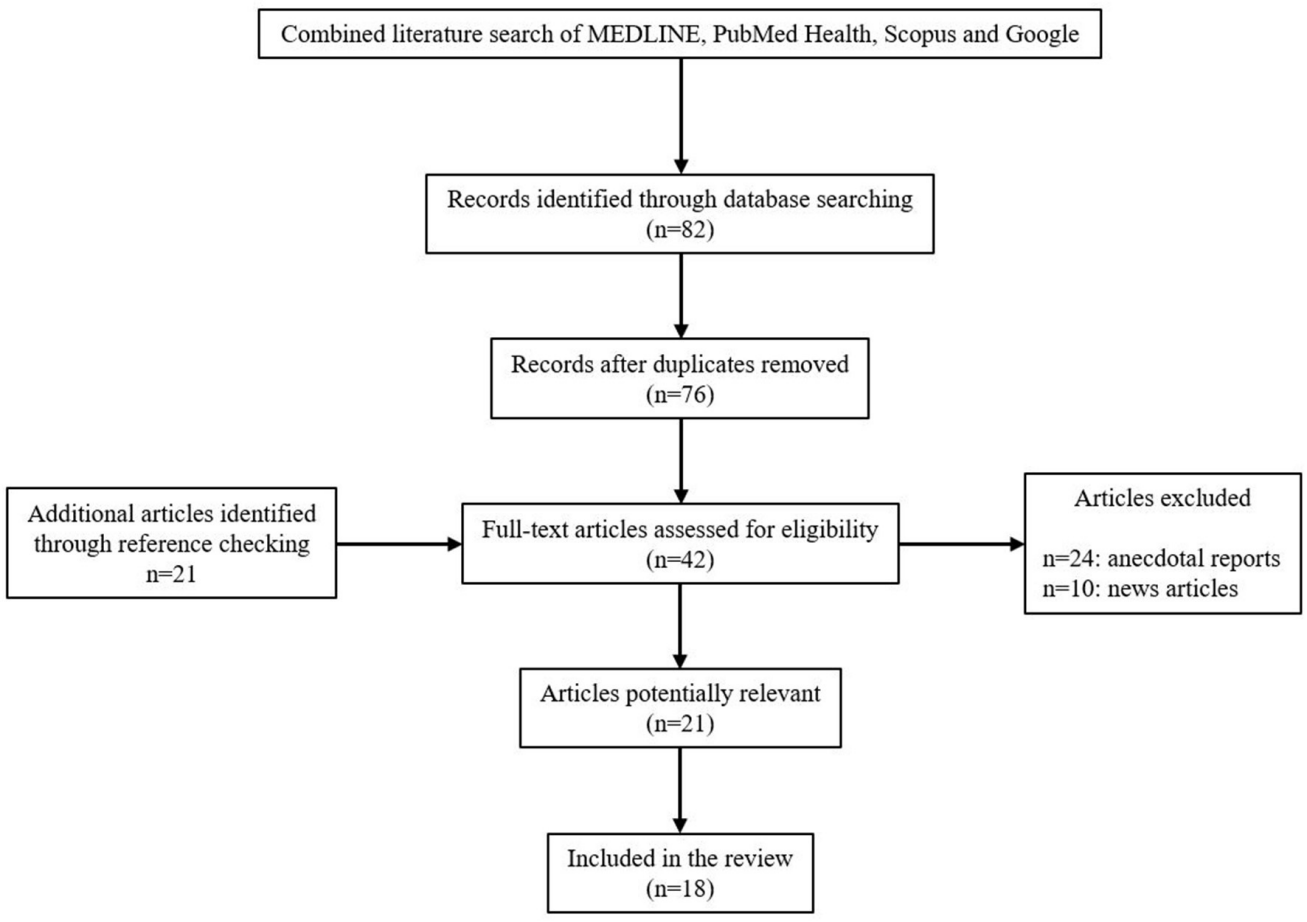
Flowchart of the review process.

A total of 18 peer-reviewed journal articles in both English and Korean languages were identified that were relevant to the present topics. A larger number of articles were identified initially but most of them were related to North Korean defectors. Those articles were excluded unless they contained object information related to the current status of the women's healthcare in North Korea (Figure 1).

### The Current Healthcare System in North Korea

**Figure 2:**
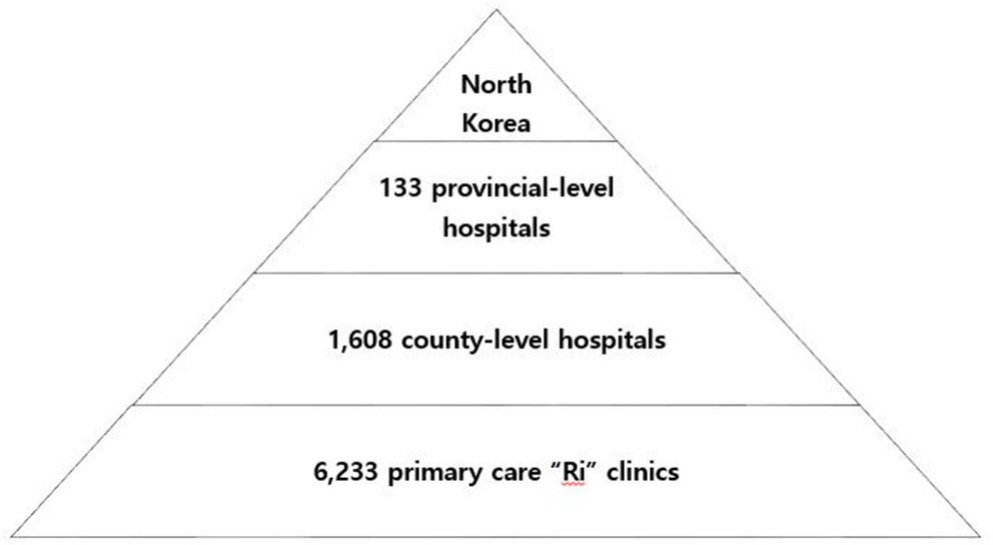
Financial aids to DPRK from South Korea. Note the fluctuating amounts of financial aids due to the political tensions between the two nations. Source: Ministry of Unification of Republic of Korea.

According to the reports by the DPRK Central Bureau of Statistics and the United Nations Children's Fund (UNICEF), there are 133 provincial-level hospitals, 1,608 county-level hospitals, and 6,233 primary care “*Ri*” clinics in North Korea (1). The estimated number of healthcare professionals including physicians, nurses, pharmacists, and midwives is about 215,000 (Table 1). The ratio of human health resources to population is the highest in the Northeastern Asia (3). “Section” or “household” doctors at the *Ri* clinics are each assigned to about 130 dwellings and are responsible for clinical and public health services, supposedly ensuring a high degree of surveillance and directly observed treatment capacity for diseases (1). *Ri* clinics link local communities to the national health system and are managed by county hospitals in the DPRK's 208 counties, which are themselves overseen by province-level referral facilities (Figure 2) (4).

**Table 1:** Comparison of the numbers of healthcare professionals between the Democratic People's Republic of Korea (North Korea) and the Republic of Korea (South Korea).

	**Democratic People's Republic of Korea (North Korea)**	**Republic of Korea (South Korea)**
Total population	24.59 million	49.55 million
Physicians	79,931	111,694
Physicians (Per 1,000 populations)	3.3	2.3
Nurses	93,400	282,846
Nurses (Per 1,000 populations)	3.9	5.6
Pharmacists	8,622	32,645
Midwives	7,368	1,051

*Source: Organization for Economic Co-operation and Development (2)*.

Despite its elaborate structure of healthcare system and abundant healthcare professionals, the actual condition its people face lags behind what the system first intended to establish. Hospitals suffer from shortages of basic items such as sterilized needles and blankets, and many have no heat, power, or running water (5). Surgeries are performed without appropriate asepsis (5). The quality of healthcare personnel including physicians, nurses, pharmacists, and midwives is not independently assessed. Healthcare workers receive basic training that emphasizes textbook learning rather than the acquisition of competency (6). There is limited supervision or access to updated knowledge, technologies, and practices (6). For many North Koreans, the main function of hospitals is to dispense medicines. Citizens lack understanding of the proper role of healthcare facilities and the importance of correct medical diagnosis (6). Under such poor circumstances, women and children in particular are more vulnerable compared to other populations. They are not receiving appropriate preventive measures of infectious disease, pregnant women do not get adequate peripartum care, and women and girls are less likely to get basic screening tests such as hemoglobin for detecting anemia.

### Obstetrical Care in North Korea

A quick glance at the statistics reported by public health organizations such as the World Health Organization (WHO) and UNICEF on women's healthcare in North Korea may not seem too dismal. The accessibility for pregnant women to healthcare facilities for antepartum services seems fairly good compared to other developing nations. According to the reports by the DPRK Central Bureau of Statistics and the United Nations Population Fund (UNFPA), 99.9% of the pregnant women between 2011 and 2014 received antepartum care. Among them, 91.8% of them received the care more than five times during one pregnancy and nearly three quarters of women received the care more than 10 times (1, 7, 8). This is greater than eight times of antenatal care visit per pregnancy recommended by the WHO (9). It was also reported that 93.9% of them received antenatal care from licensed physicians or physician assistants (8). Among the pregnant women who received antepartum care, 88.3% of them reported to check their blood pressure, 85.1% checked urinalysis, and 82.6% underwent basic blood test (8). The proportion of delivery at healthcare facilities was 91.1% and only 8.9% of pregnant women delivered at home in 2015 (8). Among the institutional deliveries, 38.3% delivered at *Ri* clinics, 35.8% delivered at county hospitals, and 17.0% delivered at province-level referral facilities (6).

Despite the sufficient number of antenatal visits to healthcare facilities and high ratio of institutional deliveries, problems exist in the actual qualities of care that are delivered. The procurement process of essential medications to manage obstetrical emergencies are poorly developed. There is a lack of accurate, consistent, and scientific methods to estimate and forecast the use of medicines (10). There is also an inadequate supply chain due to inconsistency in inventory, delay in supply leading to stock-outs, an inadequate infrastructure and lack of transportation system to healthcare facilities (10). An incorrect use of medications at healthcare facilities were also observed. Healthcare professionals were often unaware of the correct storage conditions for medications. For instance, oxytocin is a heat-sensitive medicine and must be kept at 2–8°C. According to the study in which the authors analyzed seven UNFPA and WHO reports published between 2008 and 2010, reproductive health kits, which contained oxytocin, were often stored at room temperature in many facilities (10). The cold chain requirements for this medication were also at risk due to unstable power supply or inadequate maintenance of refrigerators. Ergometrine also has restricted storage conditions. It is a light-sensitive medication, but was stored inside the delivery room, unpacked, and exposed to sunlight according to the studies (10). It was also noted that calcium gluconate, a required antidote for magnesium toxicity, was not available at all in many facilities (10). The International Federation of Gynecology and Obstetrics (FIGO) defines basic obstetrical and peripartum services as the availability of antibiotics, anticonvulsants, blood products, manual extraction of the placenta, dilatation and curettage, assisted vaginal delivery, and ability to perform basic neonatal resuscitation (e.g., with bag and mask) (11). The availability of such medical care and supplies seemed insufficient to provide adequate healthcare to the women in North Korea.

**Figure 3:**
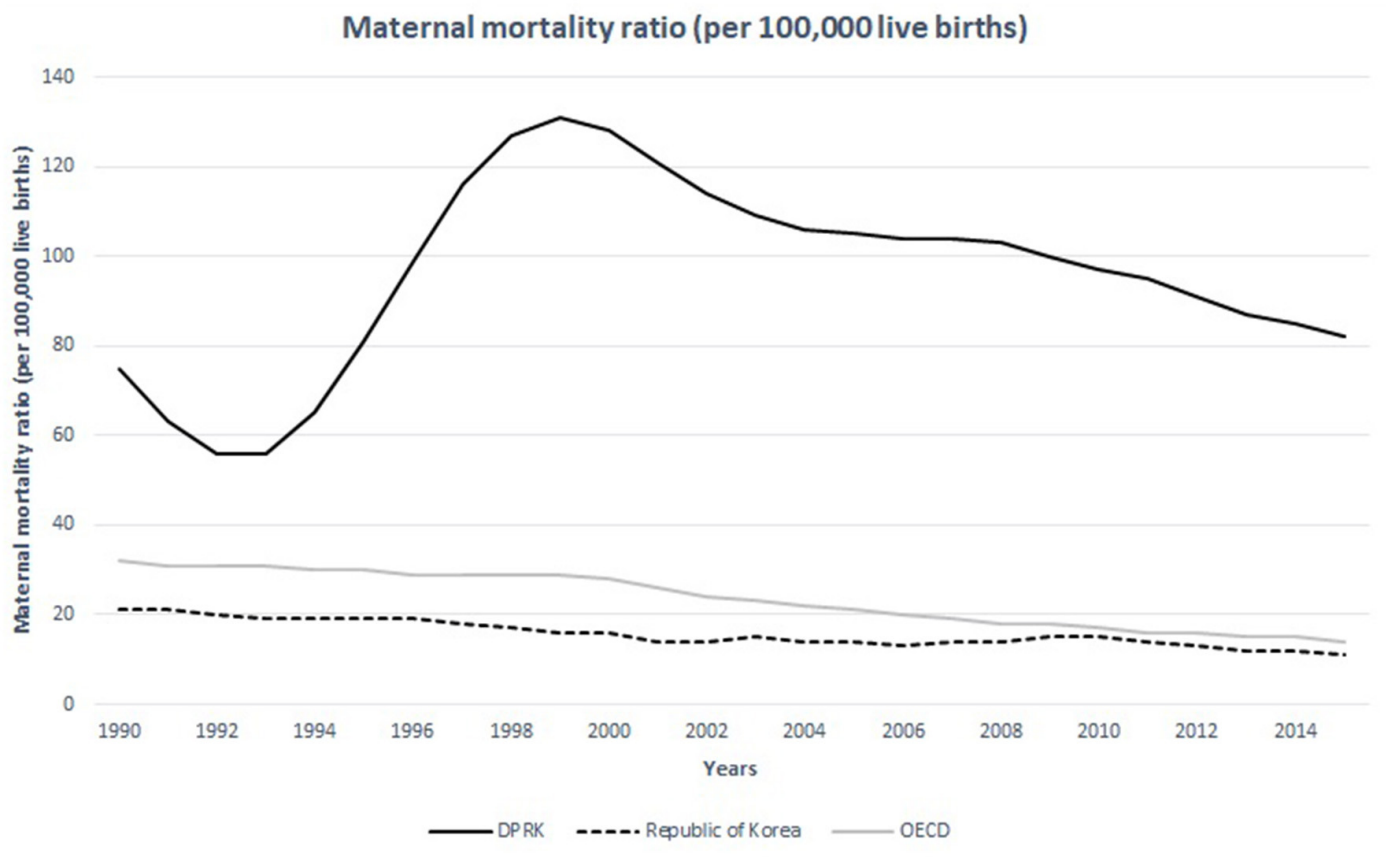
The hierarchical healthcare system of North Korea.

**Figure 4:**
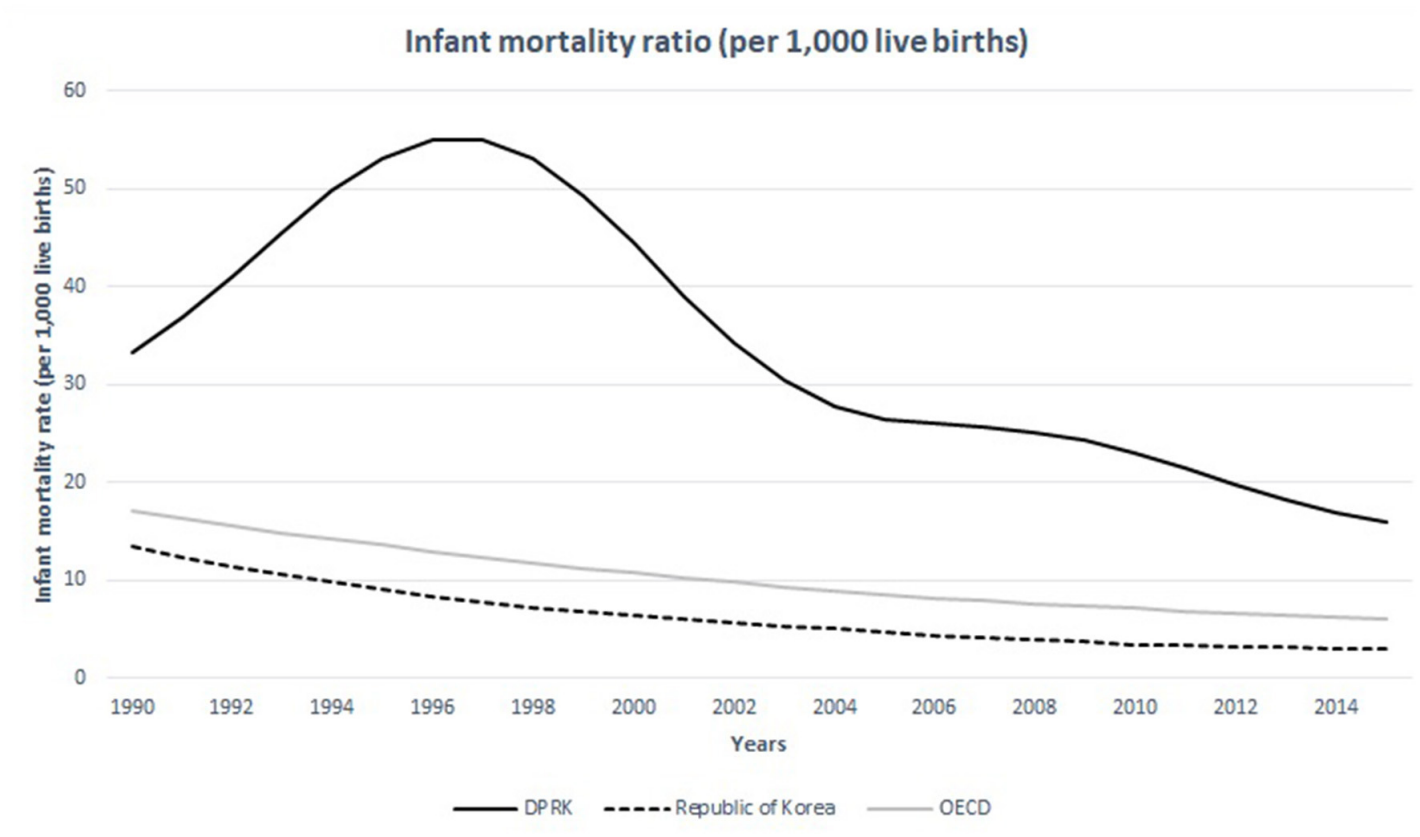
Maternal mortality ratio of the DPRK in comparison with the Republic of Korea and the nations of OECD between 1990 and 2015. Source: WHO, UNICEF, UNFPA, World Bank Group, and the United Nations Population Division (13).

Statistics mirror the poor conditions of the women's healthcare in North Korea. The maternal mortality ratio rose from 56 deaths per 100,000 live births to 82 deaths per 100,000 live births from 1993 to 2015 (Figure 3) (12). It peaked around 1999 when the devastating famine and economic crisis occurred. Despite the efforts from international communities and the UNICEF to decrease maternal mortality ratio especially since the adaptation of the eight Millennium Development Goals (MDG), the maternal mortality ratio in North Korea still seem to remain high. In 2015, the maternal mortality ratio in North Korea reached 82 deaths per 100,000 live births, almost seven times higher than that of South Korea (7). Infant mortality ratio also showed a wide gap between the two Koreas, much greater than the difference observed between Western and Eastern Germany prior to their unification (Figure 4) (14). Among 267 maternal deaths reported in North Korea in 2009, more than a third occurred in healthcare facilities (1). The leading cause of maternal death was postpartum hemorrhage, which accounted for 33% of all mothers' deaths followed by complications from abortion which accounted for 12% (7). Unsafe abortions are also an important issue regarding women's healthcare. Unsafe abortion procedures are one of the causes that contribute to high maternal mortality ratio in developing countries worldwide. It is estimated that 47,000 women die from complications of unsafe abortion in the world each year, which is about 13% of all maternal deaths (7). The government of DPRK has continuously promoted women to get pregnant in an effort to reverse the isolated nation's falling birth rate (15). The government offered tax refund and other financial benefits to encourage families to have as many children as possible (15). It has also announced birth control procedures illegal, and gynecologists who implant birth control devices in their patients are punished by law (15). The government also banned abortion procedures and imposed penalties on those healthcare providers who perform it (15). Under such circumstances, it is difficult to perform abortion procedures focusing on patient safety. Those women who experience unintended pregnancy and seek medical procedure to end it will have to undergo a dangerous illegal procedure, often jeopardizing their safety and health. It is difficult for them to obtain timely abortions, thereby reducing the risk of complications.

### Undernourished Status of the Women and Other Populations in North Korea

**Figure 5:**
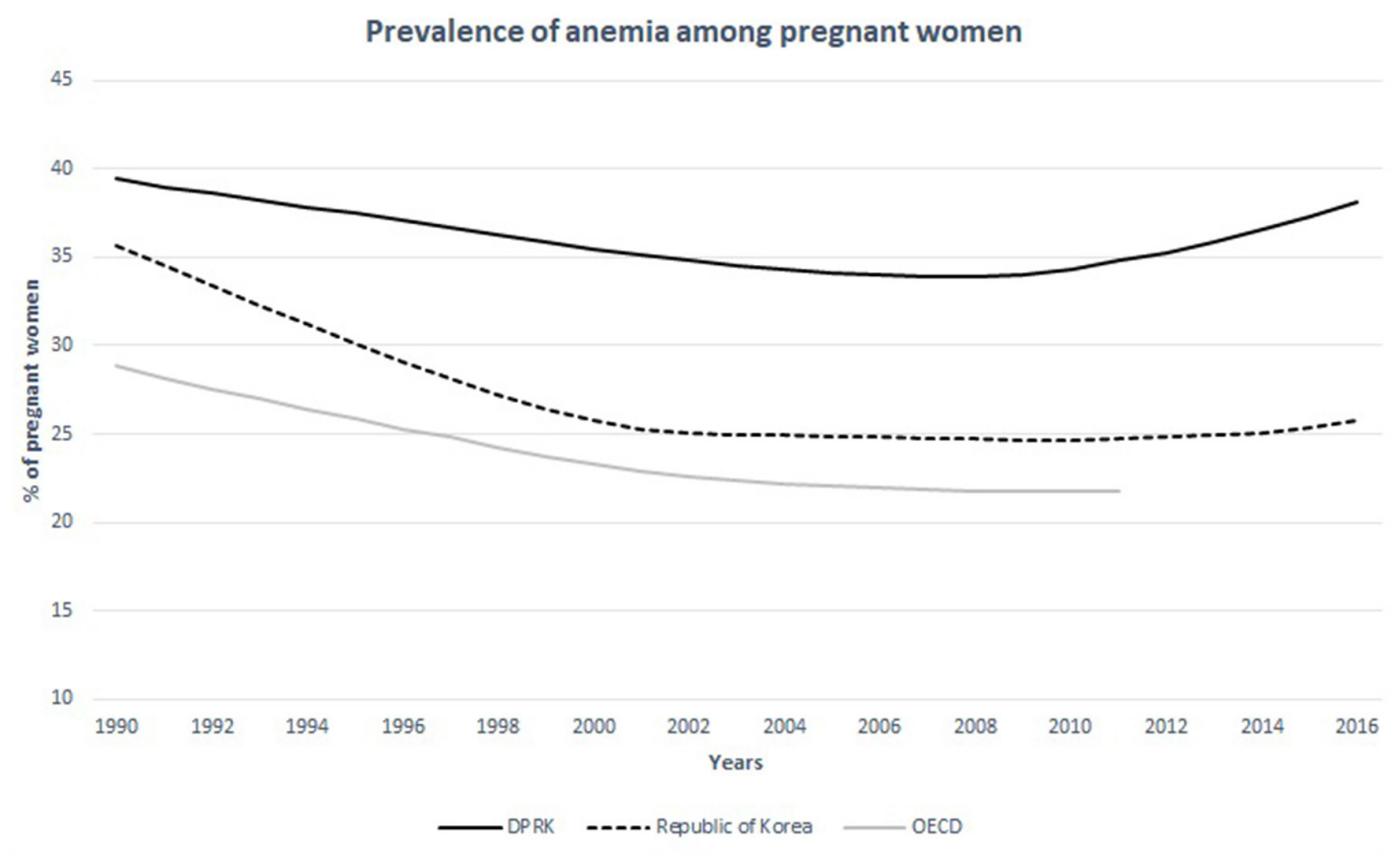
Infant mortality ratio of the DPRK in comparison with the Republic of Korea and the nations of OECD between 1990 and 2015. Source: United Nations Inter-agency Group for Child Mortality Estimation (UNICEF, WHO, World Bank, UN DESA Population Division) at childmortality.org.

**Figure 6:**
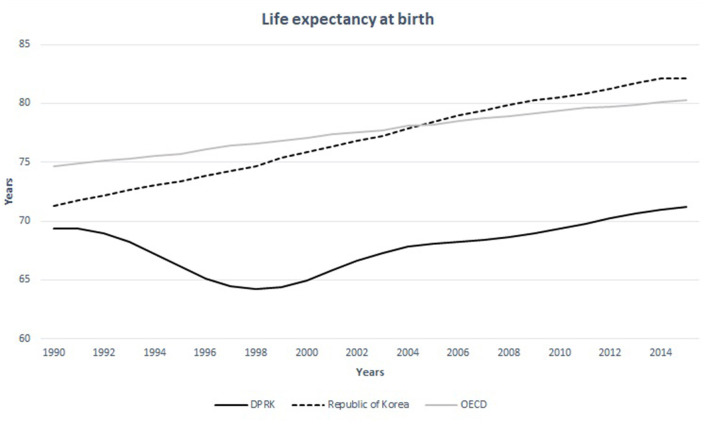
Prevalence of anemia among non-pregnant women in the DPRK in comparison with the Republic of Korea and the nations of OECD between 1990 and 2015. Source: Stevens et al. (21).

The women at child-bearing age in North Korea seem undernourished compared to other nations. The prevalence of anemia (defined by serum hemoglobin levels <12.0 g per deciliter) in women in the child-bearing age is over 30% in 15–49-year-old population while the same statistics measure 8.9, 14.2, 16.4 in 20–29, 30–39, 40–49-year-old South Korean women, respectively (Figure 5) (16, 17). The average caloric intake for an adult in North Korea is estimated at 1,314 Kcal (18). This is only the half of the calorie needs for an average adult per day in developed nations−2,450 Kcal—estimated by the United States Department of Agriculture (18). A convenient measurement that is used to assess nutritional status is the mid-upper arm circumference (MUAC). According to the UNICEF report in 2009, 25.6% of the women in North Korea between 15 and 49 years of age did not reach the MUAC of 22.5 cm, which indicated severely undernourished status (19). It has also been reported that South Korean children were 3-8 cm taller than their North Korean counterparts (20). The difference in life expectancy between the two Koreas is around 12 years for both men and women (Figure 6) (22). These life expectancy differences are much greater than the differences between Western and Eastern Germany, where the life expectancy differences were greatest around reunification (3.54 years for men and 2.95 years for women) (23).

## Discussion

The healthcare system in North Korea has achieved a substantial improvement since its division with South Korea but it is undeniable that they still remain relatively behind in the quality of care compared to other nations. The etiology of the poor delivery of healthcare in the region is multifactorial: (1) the under-prioritized status of healthcare in the government's agenda, thereby the lack of willingness by the government to address these problems, (3) the country's widespread poverty that causes insufficient basic infrastructures, and (4) the lack of understanding by its citizens of the proper roles of healthcare system in a society. In order to improve their healthcare services, we may contemplate on following issues.

Establishing a reliant and consistent way of collecting information on the healthcare system in North Korea is of paramount toward improving its healthcare function. This task must first be achieved because it can serve as the foundation for further prioritizing and solving specific issues. International organizations and the South Korean government have provided ongoing support to North Korea since 1995 (24). However, the support programs have faced several problems. The biggest problem that hinders their aids is the paucity of objective data that represent the accurate and current conditions of the healthcare system in North Korea. The donators are unsure whether the support is being distributed to those in need and this problem impedes the ability of current policy makers to plan future aid strategies. The success of healthcare systems depends on well-developed monitoring systems that include indicators to measure outcomes of financial support from international communities. The shared healthcare information between the two nations, nevertheless, should not be used politically as an instrument for regime propaganda or competition. The epidemiological research on the two Koreas should be independent from political interests.

**Figure 7:**
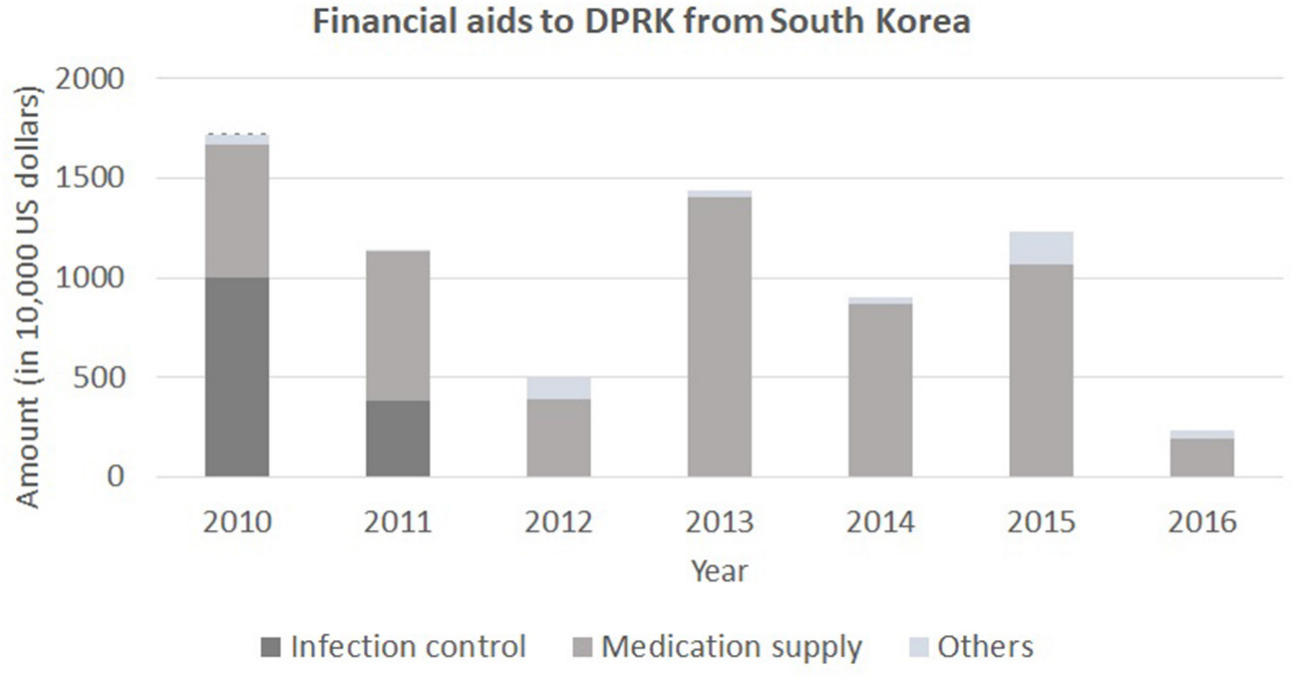
Life expectancy at birth in the DPRK in comparison with the Republic of Korea and the nations of OECD between 1990 and 2015. Source: United Nations Population Division, World Population Prospects.

Once an objective assessment of the actual conditions in North Korea is done, securing sustainable and sufficient budget that is not affected by political tensions is the next step. A wide fluctuation has been observed in the total amount of financial support to North Korea from South Korea in recent years due to the changing political conditions between the two governments (Figure 7). It is imperative to have consistent and sufficient monetary sources to develop long-term projects that are planned with clear principles without the conflict of political interests. The financial support should first be invested in the fields of pediatrics, obstetrics and infectious diseases. Equipping the basic amenities and medicines that are easy to dispense should be the initial target of international supports. Then more specialized health services such as surgery and care for chronic diseases should be provided.

Although many data sources show the healthcare system in North Korea is not self-reliant and that people are exposed to diverse problems, caution must be taken when interpreting these data. Available information regarding the health status of the nation is extremely scarce. The comments and recommendations described in the present article should therefore be interpreted cautiously and they should continuously be developed upon any new sources of available information. The two main sources of studying epidemiology of North Korea are the published reports from intergovernmental organizations and the surveys from North Korean refugees in China and South Korea. Both sources are vulnerable to biases to precisely understand the actual conditions of the nation. Reports from intergovernmental organizations may depict the actual conditions incorrectly due to the distinct nature of limitations imposed on research practiced by third-party organization, especially in a place where the provision of resources depends solely on governmental effort as a socialist system. The anecdotal reports from North Korean refugee may also distort the actual conditions of the healthcare in North Korea. Due to the limitations of available sources of information, the present study inevitably carries limitation in depicting the most accurate pictures of women's healthcare in North Korea.

While it is true that international supports have substantively improved public health in North Korea, these improvements have taken places only in selected areas and the general conditions of women's healthcare in other regions remain considerably poor. Many tasks still remain to be solved with efforts from international communities. Due to its political conditions, North Korea is in a unique position compared to other developing countries in regards to providing international health supports. They are easy to be neglected from international communities, but according to the available information so far, no one can deny the devastating conditions its people face today. It is hoped that this study increases the awareness of the professionals in women's healthcare in other regions of the world on this important issue.

## Data Availability Statement

Publicly available datasets were analyzed in this study. This data can be found here: https://www.who.int/whr/2006/whr06_en.pdf; http://apps.who.int/iris/bitstream/handle/10665/112682/9789241507226_eng.pdf;jsessionid=155319694DD66A2832B89492FE420601?sequence=2; https://www.un.org/en/development/desa/population/publications/mortality/child-mortality-report-2015.asp; https://www.who.int/publications/i/item/9789241549912; https://www.ncnk.org/sites/default/files/content/resources/publications/DPRK_NNS%20Final%20Report_%202013.pdf; https://www.data.go.kr/data/15076556/fileData.do; https://www.fao.org/3/al489E/al489e.pdf; https://data.unicef.org/country/prk/; https://www.unikorea.go.kr/eng_unikorea/news/Publications/whitepaper/.

## Author Contributions

JN collected the data and wrote the manuscript.

## Conflict of Interest

The author declares that the research was conducted in the absence of any commercial or financial relationships that could be construed as a potential conflict of interest.

## Publisher's Note

All claims expressed in this article are solely those of the authors and do not necessarily represent those of their affiliated organizations, or those of the publisher, the editors and the reviewers. Any product that may be evaluated in this article, or claim that may be made by its manufacturer, is not guaranteed or endorsed by the publisher.
